# *Ex vivo* biotransformation of lady’s mantle extracts via the human gut microbiota: the formation of phenolic metabolites and their impact on human normal and colon cancer cell lines

**DOI:** 10.3389/fphar.2025.1504787

**Published:** 2025-01-22

**Authors:** Katarzyna Jakimiuk, Aleksandra Kruk, Marta Kinga Lemieszek, Jakub W. Strawa, Sebastian Granica, Adrian Wiater, Michał Tomczyk

**Affiliations:** ^1^ Department of Pharmacognosy, Faculty of Pharmacy with the Division of Laboratory Medicine, Medical University of Białystok, Białystok, Poland; ^2^ Microbiota Lab, Department of Pharmaceutical Biology, Faculty of Pharmacy, Medical University of Warsaw, Warsaw, Poland; ^3^ Department of Medical Biology, Institute of Rural Health, Lublin, Poland; ^4^ Department of Pharmaceutical Biology, Faculty of Pharmacy, Medical University of Warsaw, Warsaw, Poland; ^5^ Department of Industrial and Environmental Microbiology, Institute of Biological Sciences, Maria Curie-Skłodowska University, Lublin, Poland

**Keywords:** *Alchemilla*, microbiota, biotransformation, colorectal cancer, deep eutectic solvent

## Abstract

**Introduction:**

For centuries, various species from the genus *Alchemilla* have been utilized in traditional medicine worldwide. Among them, *Alchemilla vulgaris* L. (Rosaceae) stands out as a promising herbal drug candidate due to its phytochemicals displaying anti-inflammatory and antioxidant properties.

**Methods:**

In our study, we investigated the interaction between the human gut microbiota and lady’s mantle herb extract (AV) following the biotransformation of the extract’s constituents and their impact on colorectal cancer cells (HT-29) and normal CCD 841 CoN epithelial cells. The *A. vulgaris* herb metabolites were obtained by incubating the extract (AV) with human fecal slurries from three healthy donors (D1, D2, and D3).

**Results:**

After incubating the AV extract with the human gut microbiota (AVD1-AVD3 samples), thirty-three metabolites were detected and characterized by LC-MS. Among them, one was identified as urolithin C. The AV and AVD1-AVD3 extracts and their metabolites exhibit various levels of antiproliferative and cytotoxic activities against cancer cells. Their biological effect might be linked to the changes and direct activity of bioavailable metabolites. Samples from AVD1, AVD2, and AVD3 increase the lactate dehydrogenase (LDH) released from damaged colon cancer cells in a dose-dependent manner. At 250 μg/mL, AVD1, AVD2, and AVD3 elevated the LDH level by 12.6%, 25.3%, and 30.0%, respectively. The biotransformed samples also showed significantly higher antiproliferative activity than the AV extract. The most active sample from donor 3 (AVD3) reached IC_50_ = 471 μg/mL.

**Discussion:**

The differences in anticancer effect might be linked to the changes and direct activity of bioavailable metabolites. The non-transformed AV extract affected neither normal nor cancer colon cells, indicating the beneficial effect of the biotransformation procedure on the anticancer properties of the evaluated extracts. The above results clearly indicate that microbial metabolism is a crucial factor that is potent in altering the biological activity of lady’s mantle extract.

## 1 Introduction

Since the Middle Ages, people have searched for natural drugs to cure their diseases. Plant-derived products play an imperative biological role against certain diseases and are a major source of modern drugs (Petrovska, 2012). Lady’s mantle (*Alchemilla vulgaris* L., Rosaceae) is a well-known plant used in folk medicine and ethnopharmacology to treat gynecological disorders such as menorrhagia, dysmenorrhea, or menstrual pain. It is distributed across temperate regions of the Northern Hemisphere, mainly in low-temperature and subarctic regions of Europe and Asia (Shilpee et al., 2021). Our recent review regarding the *Alchemilla* genus points out that *A. vulgaris* possesses a broad phytochemical composition, e.g., phenolic acids, flavonoids, anthocyanins, coumarins, and triterpenes (Jakimiuk and Tomczyk, 2024). In addition, tannins are a typical feature of the Rosaceae family, especially ellagic acid, casuarictin, pedunculagin, and agrimoniin, with documented antitumor activity (Fedotcheva et al., 2021).

A primary limitation of research investigating the pharmacological activity of polyphenols is their pharmacokinetics. Numerous scientific reports have proven that substances introduced into the body undergo metabolic changes under the influence of bacteria in the digestive tract. The produced metabolites differ in their structures and, consequently, in their biological activity from orally ingested xenobiotics (Kamada et al., 2013; Kruk et al., 2022). It is assumed the primary galenic form prepared from *A. vulgaris* is orally administered as an infusion. To prepare a single portion of the infusion, 2–4 g of the dried plant material should be added to 150 mL of hot water and left for 10 min. The recommended dosage is administered three times a day (Czygan, 2004). As mentioned, lady’s mantle extracts are a rich source of flavonoid glycosides and tannins. Currently, it is generally accepted that flavonoid glycosides are not active *per se*. Rather, these molecules are passed through further gut microbiota-mediated modification and transformation to develop their bioavailability and biological activities (Walle, 2004; Luca et al., 2020). Unabsorbed in the upper gastrointestinal tract, approximately 90% of flavonoid glycosides are subjected to enzymatic oxidation, reduction, and decarboxylation in the small intestine. Subsequently, the colon microbiota enzymatically metabolizes glycosides to aglycones, which are further transformed into ring fission products with lower molecular weights. Sulfate derivatives of these catabolites such as glucuronides produced in the liver are excreted through bile and urine (Murota et al., 2018). However, tannins are another abundant class of compounds present in *A. vulgaris*. The bioavailability of orally administrated ellagitannins and gallotannins is low mainly due to the high molecular weights of condensed polymers and interactions with other dietary elements (Piwowarski et al., 2014). The bioavailability of tannins and resulting pharmacological actions are significantly influenced by their absorption rate, possible metabolism by the gut microbiota, or liver enzymes (Serrano et al., 2009; Sallam et al., 2021). Under the influence of many gut microbiota enzymes, hydrolyzable tannins are metabolized to gallic acid, glucose (gallotannins), and ellagic acid (ellagitannins). The subsequent bacterial metabolism of ellagic acid in the colon leads to the production of urolithins which are ultimately responsible for the pharmacological effects (Espín et al., 2013).

In addition to research dedicated to identifying polyphenolic metabolites present in plants and determining their ADME parameters (absorption, distribution, metabolism, and excretion), another direction of research has been devoted to identifying the biological activity of metabolites. Due to the key role played by the gut microbiome in the metabolism of polyphenolics, colon disease therapy provides a broad spectrum for studying the pharmacological activity of secondary metabolites after the intestinal biotransformation process. Cancer of the large intestine is one of the leading causes of cancer-related death. Polyphenols have shown significant efficacy in preventing cancer development and have exhibited anticancer properties (Aiello et al., 2019). Many herbal extracts may be able to inhibit the growth and proliferation of colon cancer cells through apoptosis; cell cycle arrest in the S phase; the reduction in PI3K, P-Akt protein, and MMP expression; inhibition of DNA biosynthesis; or increase in the expression of both cell cycle inhibitors (e.g., p53, p21, p27, BAD, Bax, caspases 3, 7, 8, and 9 proteins) (El-Najjar et al., 2007; Zorofchian Moghadamtousi et al., 2014; Aiello et al., 2019). Although plant extracts display multiple anticancer effects, the clinical usage of these results requires more studies on these compounds in *in vivo* models, including bioavailability, solubility, and metabolic alteration compounds administrated *per os* or *per rectum*.

The primary objective of this study was to establish the interaction between the human gut microbiota and lady’s mantle herb extract, observing the biotransformation of the extract’s constituents and their impact on human colorectal cancer cell line HT-29 and human colon epithelial CCD841 CoN cells. Our studies create further scientific hypotheses to describe a new pharmaceutical formulation used for safe phytotherapy of gastrointestinal diseases.

## 2 Materials and methods

### 2.1 Materials

The CCD841 CoN cells were obtained from ATCC (American Type Culture Collection, Manassas, VA, United States), and the HT-29 cells were purchased from the European Collection of Cell Cultures (ECACC, Centre for Applied Microbiology and Research, Salisbury, United Kingdom). DMEM (Dulbecco’s Modified Eagle’s Medium, 41966029) supplemented with 10% FBS (fetal bovine serum, 10270106), PBS (phosphate-buffered saline, 14190136), and 100 U/mL penicillin, 100 U/mL streptomycin (15140122) were obtained from ThermoFisher Scientific (Waltham, MA, United States). The positive control 5-fluorouracil (5-FU) (343922), *In Vitro* Toxicology Assay Kit, Lactate Dehydrogenase Based (TOX7), MTT (3-(4,5-dimethyl-2-thiazolyl)-2,5-diphenyl-2H-tetrazolium bromide) (M5655), ellagic acid (E2250), and brevifolincarboxylic acid (PHL83841) were purchased from Sigma-Aldrich (Saint Louis, MO, United States). 3-*O*-caffeoylquinic and pedunculagin used as standards were isolated in the Department of Pharmacognosy Medical University of Bialystok (Poland) (Grochowski et al., 2016; Strawa et al., 2020). Methanol (MeOH) was obtained from POCH (Gliwice, Poland). Ultra-pure water was obtained in-house using a POLWATER DL3-100 deionizer (Kraków, Poland). Acetonitrile Optima (ACN) was purchased from Fisher Chemical (Loughborough, United Kingdom). The mobile phase modifier formic acid (HCOOH) was purchased from POCH (Gliwice, Poland). LC-MS analyses were conducted using an Agilent Technologies 1,260 Infinity chromatography system connected to a 6,230 time-of-flight mass spectrometer (TOF/MS) (Santa Clara, CA, United States). Experiments in anaerobic conditions were performed using a Bactron 300 Anaerobic Chamber (Sheldon Manufacturing, Inc. Cornelius, Oregon, United States).

### 2.2 Plant material

The plant material was obtained from a commercial source, Dary Natury company (Koryciny, Poland) (batch number 5902741001733). Authentication of the plant material was performed in the Department of Pharmacognosy at the Medical University of Białystok (Poland) by Michał Tomczyk, according to monography in the 11th edition of the European Pharmacopoeia (European Directorate for the Quality of Medcines and HealthCare, 2019). The examination of plant material was carried out using microscopic and thin layer chromatography (TLC) methods (European Directorate for the Quality of Medcines and HealthCare, 2019).

### 2.3 Cell cultures

The human colon epithelial cell line CCD841 CoN and the human colon adenocarcinoma cell line HT-29 were grown in Dulbecco’s Modified Eagle′s Medium/Nutrient Mixture F-12 Ham supplemented with 10% fetal bovine serum (FBS), penicillin (100 U/mL), and streptomycin (100 μg/mL). Cells were maintained in a humidified atmosphere of 95% air and 5% CO_2_ at 37°C.

### 2.4 Preparation of plant extract (AV)

The plant extract (AV) was obtained using a choline chloride-based deep eutectic solvent (DES) according to the previously described method with a slight modification (Kovač et al., 2022). In short, the DES was prepared as follows: in choline chloride/urea at a molar ratio of 1:2% and 30% of ultra-pure water (*m*/*v*). The plant material was extracted using an ultrasonic water bath (40°C, 30 min), resulting in a 20 mg/mL stock solution for evaluating the metabolism of the gut microbiota.

### 2.5 Gut microbiota metabolism of AV extract

Human fecal samples were received from three (D1, D2, and D3) healthy volunteers (28–38 years old, 2 men and 1 women) without a history of gastrointestinal disease who did not use antibiotics in the last 6 months before sample collection. All donors followed a low polyphenol diet for 3 days prior to collection. The investigations were carried out in accordance with the Declaration of Helsinki and the Ethical Committee of the Medical University of Białystok (Poland), approval no. APK.002.455.2023, allowing for feces collection from healthy human volunteers used in *ex vivo* studies. The BHI medium (brain heart infusion) was produced according to the manufacturer’s instructions by dissolving 37 g of BHI in 1 L of distilled water and then sterilizing it via autoclaving at 121°C for 15 min. All experiments with human feces were conducted under anaerobic conditions in a Bactron 300 Anaerobic Chamber. Firstly, fecal slurries were prepared by suspending human feces in BHI (1:10, *m*/*v*; 37°C) in triplicate of a particular donor. Then, 3 mL of the fecal slurries and 12.5 mL of the extract solution (20 mg/mL) were mixed with 234.5 mL of the BHI. As a control, incubations of the AV extract without fecal slurries (with BHI) and a blank without the extract in the BHI (fecal slurries with the BHI) were carried out. Prepared samples were incubated in an anaerobic condition for 24 h. After incubation, the metabolic reactions were terminated by adding MeOH–0.1% HCOOH (1:1, *v*/*v*) to 1 mL of each sample before being sent for LC/MS analysis of the obtained metabolites. The remaining mixtures were centrifuged and fractionated via solid phase extraction (SPE) using 30% H_2_0% and 100% MeOH. The obtained fractions (AVD1-AVD3) were evaporated, lyophilized, and used for biological analysis. All samples from which we could obtain biological material but which were not used for further analysis were destroyed.

### 2.6 LC-MS analysis of AV and AVD1-AVD3

The AV extract and AVD1-AVD3 samples were analyzed using an adapted and slightly modified method developed by Duckstein and co-workers (Duckstein et al., 2012). A Poroshell 120 EC-C18 column measuring 250 mm × 2.1 mm, and 4 mm was used to adapt the conditions of chromatographic separation. Chromatographic analyses were carried out using an Agilent Infinity 1260 LC system equipped with a vacuum degasser, a binary pump, an autosampler, a thermostatic column compartment, and a PDA detector (Agilent, USA). An analytical reverse-phase column was used at a temperature of 25°C with detection wavelengths of 280 and 360 nm to identify tannins and flavonoids, respectively. The eluents were 0.1% HCOOH (eluent A) and ACN/H_2_O (9:1, *v*/*v*; eluent B) used for the following gradient with a constant flow rate of 0.350 mL/min: 0–5 min, 0% B; 5–40 min, 0%–12.5% B; 40–105 min, 12.5%–25% B; 105–110 min, 25%–100% B; 110–115 min, 100% B; and then re-equilibration to starting conditions. The injection volume was 10 μL. TOF/MS analyses were performed using an Agilent 6,230 mass spectrometer with an ESI ion source. MS acquisition was performed under the following conditions: negative and positive ionization mode with capillary voltages of 2,500 and 4000 V, respectively; dry gas flow (N2), 9 L/min; nebulizer pressure, 35 psi; capillary temperature, 365°C. Mass spectra were recorded between m/z 50 and 2000. Peaks were identified according to their specific fragmentation patterns, UV spectra, and retention times compared with literature data and commercial reference standards. The MassHunter Qualitative Analysis V 10.0 software (Agilent) was used for LC-MS control and data processing.

### 2.7 Preparation of samples for anticancer activity evaluation

Stock solutions of investigated samples (AV, AVD1-AVD3) (20 mg/mL), were prepared by dissolving them in the DMSO/H_2_O (1:1, *v*/*v*) mixture and were stored in the fridge. Working solutions of the investigated samples were prepared by dissolving an appropriate stock solution in a culture medium. The working solutions were prepared to contain the same amount of the solvent mixture. As a control of the experiment, 25 μg/mL 5-fluorouracil (5-FU) was used and dissolved in the DMSO/H_2_O (1:1, *v*/*v*) mixture.

### 2.8 Assessment of compounds cytotoxicity–lactate dehydrogenase (LDH) assay

The cells were seeded in 96-well microplates at a density of 5 × 10^4^ cells/mL. The following day, the culture medium was removed, and the cells were exposed to the investigated samples (AV, AVD1-AVD3) prepared in a fresh medium supplemented with 2% FBS. After 96 h of incubation under standard conditions (5% CO_2_, 37°C), the culture supernatants were collected in new 96-well microplates, which were used to perform the LDH assay following the manufacturer’s instructions (*in vitro* Toxicology Assay Kit Lactate Dehydrogenase Based). The test was based on measuring the LDH released into the culture medium upon damage to the cell plasma membrane. Absorbance readings were recorded using a microplate reader (BioTek ELx800, Highland Park, Winooski, Vermont, United States) at a 450 nm wavelength. The results are presented as the percentage of LDH released from cells treated with the tested compound versus cells grown in the control medium (indicated as 100%).

### 2.9 Assessment of cell proliferation–MTT assay

The cells were seeded on 96-well microplates at a density of 5 × 10^4^ cells/mL. On the following day, the culture medium was removed, and the cells were exposed to the investigated samples (AV, AVD1-AVD3) prepared in a fresh medium supplemented with 10% FBS. After 96 h of incubation, under standard conditions (5% CO_2_, 37°C), the MTT solution (5 mg/mL in PBS) was added to the cells for 3 h. The resultant crystals were solubilized overnight in SDS buffer with a pH of 7.4 (10% SDS in 0.01 N HCl), and the product was quantified spectrophotometrically by measuring the absorbance at a 570 nm wavelength using a microplate reader (BioTek ELx800, Highland Park, Winooski, Vermont, United States). The results are presented as a percentage of the metabolic activity of cells treated with the investigated compound versus cells grown in the control medium (indicated as 100%).

### 2.10 Statistical analysis

The data were presented as mean ± SEM. One way ANOVA with Tukey’s *post hoc* test and column statistics were used for comparisons. Significance was accepted at *p* < 0.05. The IC_50_ value (concentration leading to 50% inhibition of proliferation compared to the control) was calculated using GraphPad PRISM.

## 3 Results

### 3.1 Composition of the AV extract and metabolized samples (AVD1-AVD3)

**FIGURE 1:**
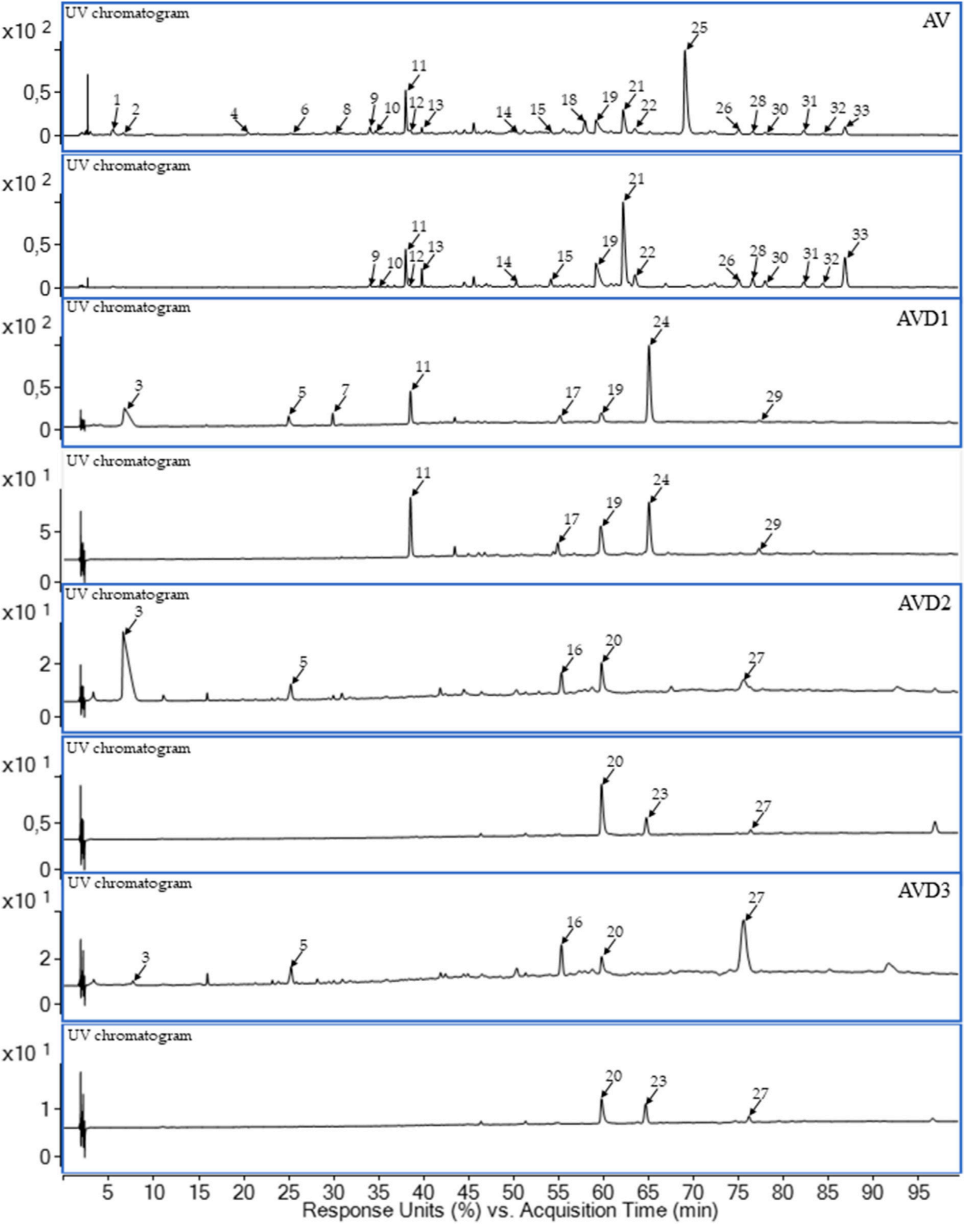
The LC-PDA-ESI-TOF/MS extracted ultra-violet chromatograms (UV) of representative samples of Lady’s mantle herb DES extract (AV) donors’ samples (AVD1-AVD3) after 24 h of incubation with human gut microbiota. Upper line – 280 nm, lower line – 365 nm; D1–D3 – faecal donor number.

In the first step of our experiment, we conducted LC-PDA-ESI-TOF/MS analysis of the raw AV extract. As shown in Figure 1 and Table 1, the use of a eutectic mixture led to the extraction of thirty three compounds, including flavonoid derivatives (e.g., quercetin and kaempferol *O*-glucuronides) and tannins (e.g., agrimoniin, pedunculagin α or β, and sanguiin H-10 isomer). The study of postbiotic metabolites resulted in the detection of twelve metabolites, where three were identified as brevifolincarboxylic acid (**11**), ellagic acid (**19**), and urolithin C (**24**) (donor D1) (Figure 1; Table 1). It is worth noting that both the crude extract and the metabolized samples contained **11** and **19**. The presence of **19** among the products of biotransformation by the intestinal gut microbiota is proof of the degradation of ellagitannins and the gradual breakdown process before the formation of urolithins. Urolithin C (**24**), absent from the AV extract, was detected in the metabolized sample (AVD1). The presence of several unclassified metabolites (**3**, **5**, **7**, **16**, **20**, **23**, **27**, **29**) in the donor samples and their absence in the AV extract indicate that a biotransformation process of plant secondary metabolites had occurred.

**TABLE 1:** The UV–Vis and MS data of compounds identified in AV and AVD1–AVD3 by LC-PDA-ESI-TOF/MS.

No.	Rt [min]	UV spectra [λ max nm]	Observed^a^	Δ [ppm]	Formula	Fragmentation		Tentatively predicted compounds	Extract/samples			
						Negative ionisation	Positive ionisation		AV	AVD1	AVD2	AVD3
1	5.36	316	182.99276	3.3	C_7_H_4_O_6_	366, **182**, 139	-	2-Pyrone-4,6-dicarboxylic acid isomer^c^	+	-	-	-
2	6.31	316	182.99422	3.86	C_7_H_4_O_6_	366, **182**, 139	-	2-Pyrone-4,6-dicarboxylic acid isomer^c^	+	-	-	-
3	6.38	275	233.10194	−4.71	C_10_H_18_O_6_	**233**, 153	**235**	Unknown	-	+	+	+
4	20.46	260*sh*	783.0718	4.05	C_34_H_24_O_22_	**783**, 481, 301	-	Pedunculagin α or β (s)^c^	+	-	-	-
5	24.94	272	834.41302	2.35	C_36_H_67_O_21_	**834**, 203	**836**, 205	Unknown	-	+	+	+
6	26.17	295*sh*, 325	353.08747	−0.96	C_16_H_18_O_9_	**353**, 191, 179	355, **163**	5-*O*-caffeoylquinic acid (s)	+	-	-	-
7	29.83	225*sh*, 275	529.25755	−3.58	C_33_H_37_O_6_	**529**, 259, 243	-	Unknown	-	+	-	-
8	30.00	260*sh*	783.072	4.26	C_34_H_24_O_22_	**783**, 481, 301	-	Pedunculagin α or β (s)^c^	+	-	-	-
9	33.98	295*sh*, 325	353.0892	4.01	C_16_H_18_O_9_	**353**, 191	355, **163**	3-*O*-caffeoylquinic acid (s)^c^	+	-	-	-
10	34.52	230, 315	325.09416	3.92	C_15_H_17_O_8_	**325**, 145	**250**, 121	Unknown	+	-	-	-
11	34.63	278, 360	291.016	4.55	C_13_H_8_O_8_	**291**, 247	293	Brevifolincarboxylic acid (s)	+	+	-	-
12	35.09	275	633.07359	0.39	C_27_H_22_O_18_	**633**, 463, 301	-	Galloyl-HHDP-glucose^c^	+	-	-	-
13	39.73	270, 350	639.12498	5.63	C_27_H_28_O_18_	**639**, 463, 301	**641**, 465, 303	Quercetin *O*-hexoso *O-*uronic acid derivative	+	-	-	-
14	50.22	254, 268sh, 348	637.10943	−1.69	C_20_H_29_O_23_	637	**639**, 287	Flavonol derivatives	+	-	-	-
15	54.11	255, 268*sh*, 295*sh*, 355	623.12565	0.45	C_27_H_27_O_17_	**623**	**625**, 303	Quercetin derivatives	+	-	-	-
16	55.22	280	1445.72706	0.33	C_78_H_109_O_6_	**1,445**, 929, 282	**1,447**, 931, 284	Unknown	-	-	+	+
17	55.38	274	596.33914	−3.74	C_28_H_53_O_13_	**596**	**598**, 284	Unknown	-	+	-	-
18	57.91	255	1567.15152	−0.56	C_68_H_48_O_44_	1,567, 1,265, **783**, 301	-	Sanguiin H-10 isomer^c^	+	-	-	-
19	59.20	254, 370	300.99841	−1.6	C_14_H_6_O_8_	**301**, 271	**303**	Ellagic acid (s)^c^	+	+	-	-
20	60.36	254, 367	1090.5504	−0.76	C_60_H_82_O_18_	**1,090**, 300	**1,092**	Unknown	-	-	+	+
21	62.19	256, 354	477.06713	−0.6	C_21_H_18_O_13_	**477**, 301	**479**, 303	Quercetin *O*-glucuronide^c^	+	-	-	-
22	63.40	254, 266*sh*, 348	461.0742	3.57	C_21_H_18_O_12_	**461**, 285	**463**, 287	Kaempferol *O*-glucuronide	+	-	-	-
23	64.81	264, 352, 400*sh*	836.42674	0.77	C_36_H_68_O_21_	**836**, 299	**838**, 301	Unknown	-	-	+	+
24	65.05	258, 306, 338	243.02980	−0.09	C_13_H_8_O_5_	**243**	**245**	Urolithin C	-	+	-	-
25	68,99	230, 260	1870.15790	−0.41	C_82_H_54_O_52_	1870, 1,567, 1,246, **934**, 783, 301	-	Agrimoniin^c^	+	-	-	-
26	75.00	231, 266, 336	445.07909	3.28	C_21_H_18_O_11_	**445**, 269	**447**, 271	Flavone derivative	+	-	-	-
27	75.55	270	687.41612	−1.61	C_32_H_64_O_15_	**687**	**689**, 461, 345	Unknown	-	-	+	+
28	76.55	252, 356	491.08441	2.64	C_22_H_20_O_13_	**491**, 315	**493**, 317	Methyl quercetin glucuronide^c^	+	-	-	-
29	77.31	252, 360	1077.60771	0.89	C_50_H_94_O_24_	1,077	1,079, 758, 689, 345	Unknown	-	+	-	-
30	84.33	266, 350	477.10284^b^	0.02	C_22_H_20_O_12_	951, **475**, 299	**477**, 300	Flavonol derivative	+	-	-	-
31	82.18	258, 273, 343	523.10790	1.23	C_23_H_22_O_14_	**521**, 345	**523**, 431, 347, 269	Flavonoids derivative	+	-	-	-
32	84.33	266, 350	477.10284^b^	0.52	C_22_H_20_O_12_	951, **475**, 299	**477**, 300	Flavonol derivative	+	-	-	-
33	86.76	254, 273*sh*, 355	505.09952	2.08	C_23_H_21_O_13_	1,011, **505**, 329	**507**, 331	Unknown	+	-	-	-

^a^ Exact mass of [M-H]- ion.

^b^ Exact mass of [M-H]+ ion; sh, peak shoulder; bold, most aboundantion; s, reference substance; HHDP, hexahydroxydiphenoyl group.

^c^ Correlated with published data (Duckstein et al., 2012).

### 3.2 Anticancer activity evaluation

**FIGURE 2:**
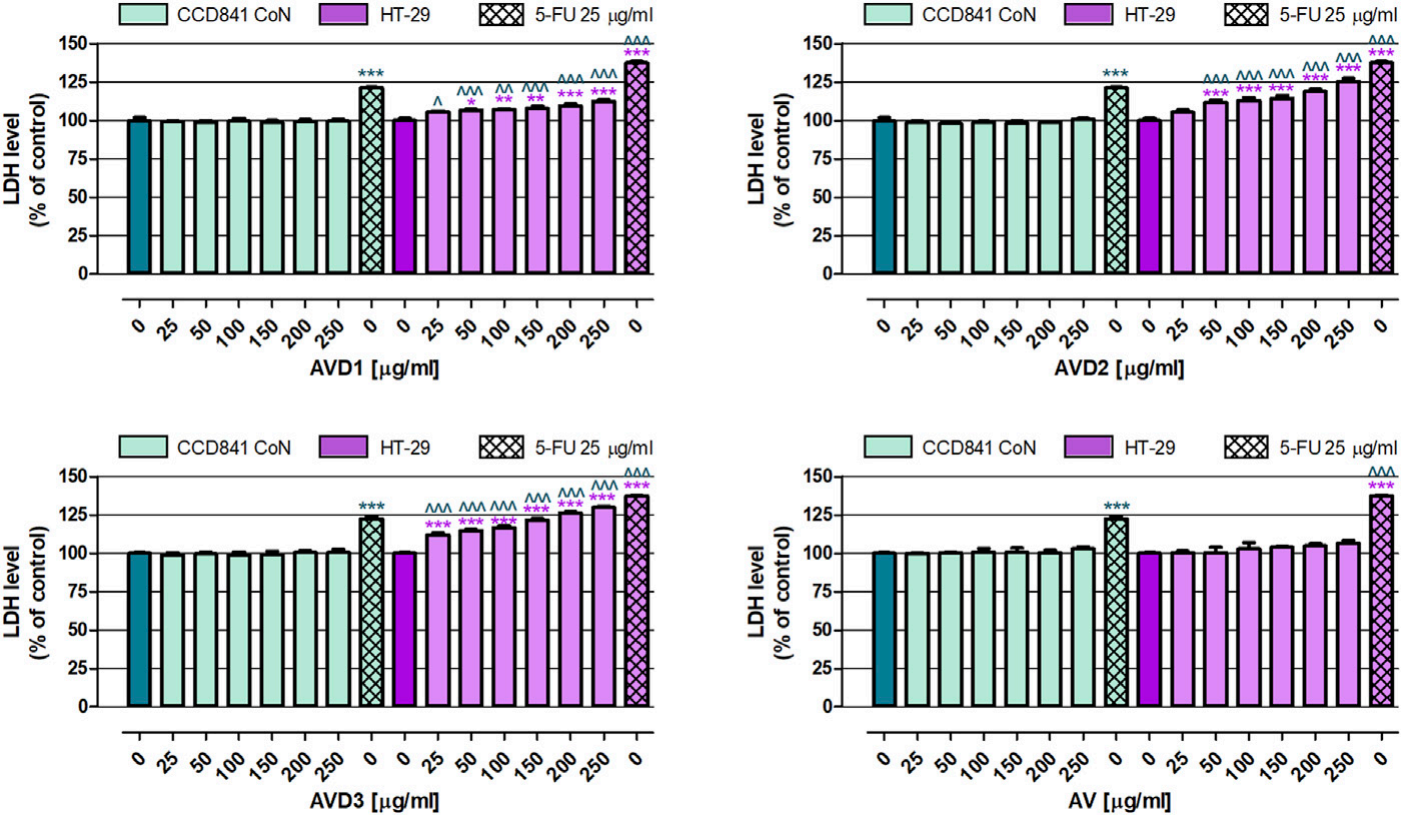
Influence of the *Alchemilla vulgaris* extract (AV) and samples after biotransformation (AVD1-AVD3) on cell membrane integrity of human colon epithelial cell line CCD841 CoN and human colon adenocarcinoma cell line HT-29. The cells were exposed to the culture medium alone (control) or extracts at concentrations of 25, 50, 100, 150, 200 and 250 μg/mL; or 25 μg/mL, 5-fluorouracil (5-FU; positive control) for 96 h. Samples cytotoxicity (level of LDH released into the cell culture medium from damaged cell membranes) was measured using an LDH assay. Results are presented as mean ± SEM of 6 measurements. *p < 0.05; **p < 0.01; ***p < 0.001 vs. control; ^∧^p < 0.05; ^∧^^∧^p < 0.01; ^∧^^∧^^∧^p < 0.001 colon cancer cells treated with extract/5-FU vs. colon epithelial cells exposed to the extract/5-FU at the corresponding concentration; one-way ANOVA test; post-test: Tukey.

In the first set of anticancer experiments, the cytotoxicity of samples was examined in both normal and cancer colon cell lines. As shown in Figure 2, these all investigated extracts (AV, AVD1-AVD3) in the whole range of tested concentrations (25–250 μg/mL) did not affect the membrane integrity of the human colon epithelial cell line CCD841 CoN, while, when used as a positive control of the experiment, 25 μg/mL 5-FU increased the LDH level to an average of 121.8%. Studies on the human colon adenocarcinoma cell line HT-29 revealed that the AV extract in the whole range of tested concentrations was not cytotoxic. On the contrary, AVD1, AVD2, and AVD3, in a dose-dependent manner, increased LDH released from damaged colon cancer cells. Of the samples that underwent biotransformation, the strongest cytotoxic effect induced AVD3, which, at the higher tested concentration, elevated the LDH level by 30.0%, while AVD1 and AVD2 decreased the membrane integrity of cancer cells by 12.6% and 25.3%, respectively. On the contrary, 25 μg/mL 5-FU increased the LDH level to an average of 137.3%. It must be highlighted that AVD1 and AVD2 have shown cytotoxic properties against HT-29 in concentrations ranging from 50 to 250 μg/mL, while AVD3 in the whole range of tested concentrations significantly damaged the membranes of colon cancer cells. Comparison data collected from normal and cancer cells treated with biotransformed extracts revealed their great selectivity, indicating that compounds were not toxic against CCD841 CoN cells and, at the same time, significantly decreased the viability of HT-29 cells. The non-transformed AV extract did not affect either cancer or normal colon cell lines.

**FIGURE 3:**
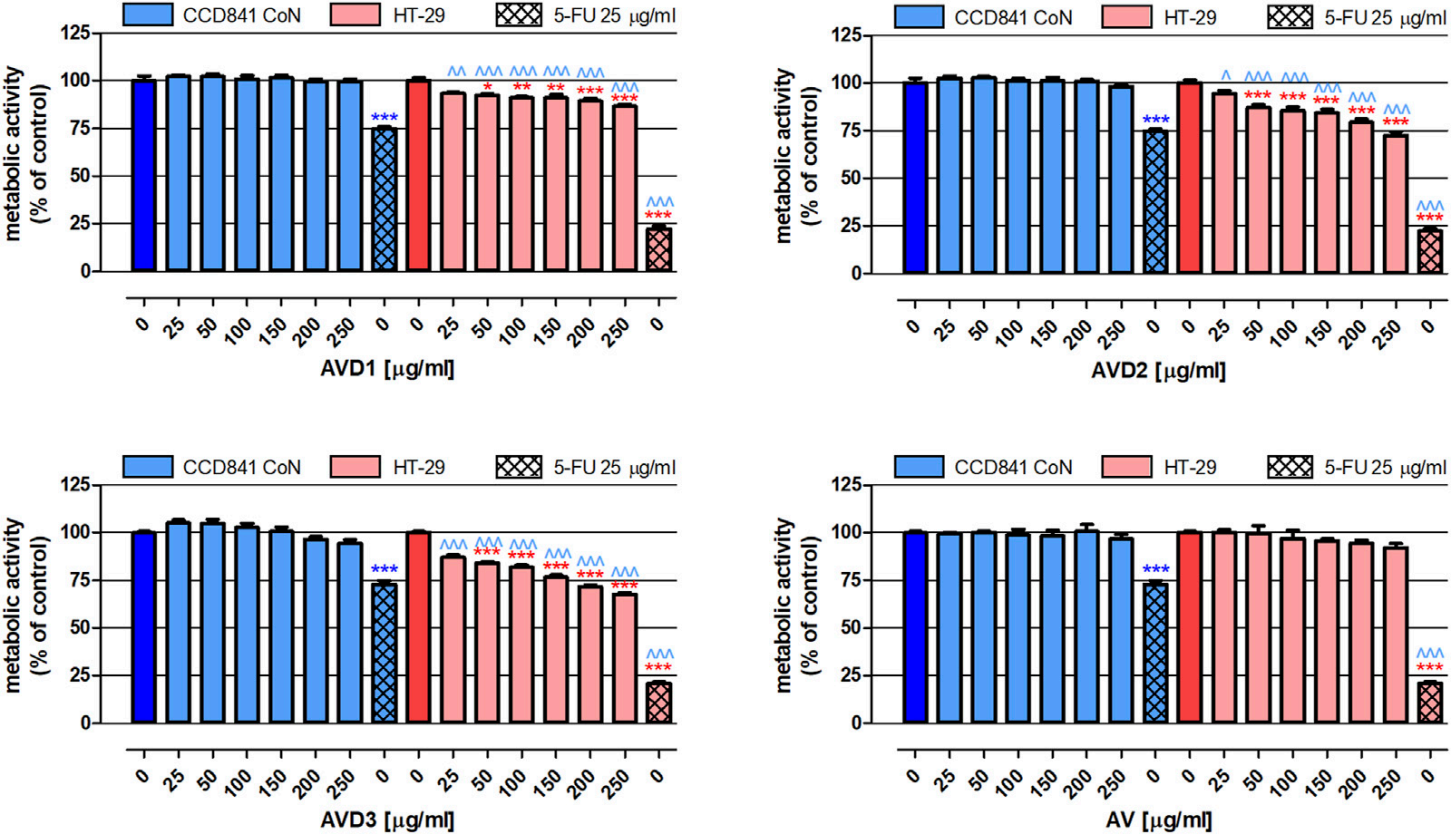
Influence of the *Alchemilla vulgaris* extract (AV) and samples after biotransformation (AVD1-AVD3) on the proliferation of human colon epithelial cell line CCD841 CoN and human colon adenocarcinoma cell line HT-29. The cells were exposed to the culture medium alone (control) or extracts at concentrations of 25, 50, 100, 150, 200 and 250 μg/mL; or 25 μg/mL 5-fluorouracil (5-FU; positive control) for 96 h. The metabolic activity of investigated cells in response to tested compounds was examined photometrically by means of the MTT assay. Results are presented as mean ± SEM of 6 measurements. *p < 0.05; **p < 0.01; ***p < 0.001 vs. control; ^∧^p < 0.05; ^∧^^∧^p < 0.01; ^∧^^∧^^∧^p < 0.001 colon cancer cells treated with extract/5-FU vs. colon epithelial cells exposed to the extract/5-FU at the corresponding concentration; one-way ANOVA test; *post hoc* test: Tukey.

In the next step of our study, the extracts’ impact on the proliferation of both normal and cancer colon cells was examined. As presented in Figure 3, none of the tested extracts altered the metabolic activity of human colon epithelial cell line CCD841 CoN. Moreover, AV did not impact the proliferation of human colon cancer HT-29 cells. On the contrary, AVD1- AVD3 in concentrations ranging from 50 to 250 μg/mL revealed significant antiproliferative properties in HT-29 cells, and the observed changes were dose-dependent. Among biotransformed extracts, the strongest inhibition (by 32.2%) of cancer cell proliferation was observed after treatment with 250 μg/mL AVD3 (IC_50__HT-29_ = 471 μg/mL), while the weakest effect was observed for AVD1 (IC_50__D1 HT-29_ = 1,440 μg/mL), which, at the concentration of 250 μg/mL, reduced metabolic activity by 13.4%. Used as a positive control, 25 μg/mL 5-FU decreased the proliferation of CCD841 CoN and HT-29 cells by 26.1% and 78.4%, respectively.

## 4 Discussion

*Alchemilla vulgaris* is one of the most well-known medicinal plants in terms of its phytochemical content. According to recent research, deep eutectic solvents (DESs) have been adapted for new methods to extract and isolate polyphenols (Zainal-Abidin et al., 2017). The main benefits of the use of DESs are the low cost of the starting components and their biodegradable properties with no or low toxicity. The selection of the DES for our experiments was based on an examination conducted by Kovač and co-workers. According to their article, choline chloride/urea (1:2, molar ratio) was the most favorable mix of components due to the highest yields of gallic acid, ellagic acid, and hydrolyzable tannins. As the authors mentioned, the level of desired components was also affected by the amount of added water due to the impact on the viscosity that affects mass transfer in the extraction process (Kovač et al., 2022). The following phytochemicals are prevalent in the literature: pedunculagin isomers (**4**, **8**), brevifolincarboxylic acid (**11**), sanguiin H-10 isomer (**18**), ellagic acid (**19**), and agrimoniin (**25**). These phytochemicals are found in ethanol and acetone/water extracts from *A. vulgaris* and are also present in DES extracts (Duckstein et al., 2012). Thus, in the crude *A. vulgaris* extract (AV), numerous compounds from the polyphenol group were present, contains specific molecules belonging to a large group of tannins (Table 1).

Ten compounds were detected, which were not observed in the crude extract (AV). Even though most of the reported examinations agreed on the resulting products of the digestion of plant secondary metabolites, the qualities and quantities of these metabolites may vary among individuals. These differences may relate to variations in the composition of the colonic microbiota, dietary habits, and health status among individuals. Ion fragmentation analysis results show that they are formed by ellagitannins. In addition to the pattern-compliant retention time, the ion distribution confirms the presence of the alpha and beta isomers of pedunculagin (**4**, **8**) in the extract. We have observed ions at m/z 783 and m/z 481 in the MS spectra, indicating the loss of HHDP (hexahydroxydiphenoyl unit), and at m/z 301, indicating the loss of hexose + O. It is worth noting that in the ion distribution of compound **25**, common elements were observed. The initial phase of MS fragmentation involves the cleavage of two HHDP units at m/z 1,567 (loss of HHDP) and m/z 1,265 (loss of HHDP), which are distinguished by a GOG-type bond between the two galloyl groups, comprising compound **25** (with two galloyl-bis-HHDP hexose units). Subsequently, **25** has been cleaved at m/z 783 (loss of 132 Da + loss of HHDP) and m/z 301 (loss of hexose + O). The loss of 132 Da from a galloyl moiety without oxygen after cleavage showed that the type of linkage was a GOG. In the present investigation, the fragmentation of the dimeric ellagitannin structure (m/z 1,567 → m/z 783 → m/z 301) led to the conclusion that this ellagitannin was sanguiin H-10 (**18**).

Traditionally, lady’s mantle is administered orally and thus undergoes intestinal digestion. Although this plant material occurs in the pharmacopeial monograph, there are no reports on its interaction with the human gut microbiota (ESCOP, 2003). Data from the literature indicate that ellagic acid can be transformed by the gut microbiota into urolithin C (Kujawska and Jodynis-Liebert, 2020). According to the information in Table 1, ellagic acid (**19**, m/z = 302) present in the AV extract underwent biotransformation, as it is not present in the samples from donors. This fact is also confirmed by the occurrence of urolithin C (**24**, m/z = 244) in the donor D1 sample (AVD1 after the biotransformation process). Another large group of phytochemicals present in the AV extract but missing in donor samples (AVD1-AVD3) are flavonoids and their derivatives. Their gut microbiota-mediated biotransformation depends on the attached moiety (Murota et al., 2018; Al-Ishaq et al., 2021). As flavonoid derivatives, when they reach the colon, they can be hydrolyzed to aglycons and into ring fission products (Pei et al., 2020; Xiong et al., 2023). Thus, quercetin and kaempferol *O*-glucuronides occur in the AV extract but they are lacking in the metabolized donor samples (AVD1-AVD3). The compounds present in the AV extract may have undergone complete degradation or may be the compounds marked as unidentified (Table 1).

As mentioned in the introduction, biotransformation through the microbiota and modifications in the composition of lady’s mantle may influence its therapeutic properties and qualities. At this point, we evaluated the anticancer potential of samples after biotransformation compared to the crude extract (AV). So far, the anticancer potential of *A. vulgaris* extracts has been proven in the murine melanoma cell line B16, human ovarian cancer cell line A2780, human prostate cancer cell line PC-3, and human breast cancer cell lines 4T1 and MCF-7 (Vlaisavljević et al., 2019; Jelača et al., 2024; Al-Zharani and Abutaha, 2023). There is also a scientific report presenting the efficacy of a water/methanol extract (2:8, *v*/*v*) of *A. vulgaris* against human colorectal adenocarcinoma cell line Caco-2 (Ibrahim et al., 2022). In the presented study, we also focused on human colorectal cancer cells; however, the investigation also included an assessment of the extracts’ impact on normal human colon epithelial cells, which, unfortunately, is not a very common procedure. The performed analysis revealed that the significant chemopreventive potential of *A*. *vulgaris* extracts after biotransformation (AVD1, AVD2, and AVD3) manifested via the effective elimination of human colon cancer HT-29 cells. This caused both damage to their cell membranes (LDH assay) and inhibition of their proliferation (MTT assay), with the simultaneous lack of cytotoxicity and antiproliferative impacts on normal colon epithelial CCD841 CoN cells (Figures 2, 3). The obtained data indicate the high selectivity of the tested extracts, which is crucial for their potential therapeutic use. An extremely valuable observation is that the AV extract did not cause any changes in both normal and cancer cell lines, indicating that biotransformation is required to reveal the beneficial health-promoting properties of the examined extracts. In addition, the donor samples are a post-fermentation mixture of various compounds created from the culture medium and microbial metabolism, which can mutually interact with the used model.

## 5 Conclusion

*Alchemilla vulgaris* extract contains phytoconstituents from the group of polyphenols, the largest group of which are flavonoids and tannins. After gut microbiota biotransformation, there were changes in the chemical composition of the donors’ samples compared to the native extract. Our study revealed the great anticancer potential of biotransformed *A. vulgaris* extracts, which selectively eliminated human colon cancer HT-29 cells without any negative changes in human colon epithelial CCD841 CoN cells. The lack of changes in the viability and proliferation of the investigated normal and cancer cell lines in response to non-transformed *A. vulgaris* extract clearly indicates the beneficial effect of the biotransformation procedure on the anticancer properties of evaluated extracts. However, the exact mechanism of these interactions remains undiscovered and requires further investigations of microbiota composition changes and direct action of bioavailable metabolites. Our finding indicated new therapeutic possibilities for *A. vulgaris* extract in the context of gastrointestinal diseases and interesting concepts of the composition of potential drugs of natural origin used in safe phytotherapy.

## Data Availability

The raw data supporting the conclusions of this article will be made available by the authors, without undue reservation.
